# A flexible and wearable NO_2_ gas detection and early warning device based on a spraying process and an interdigital electrode at room temperature

**DOI:** 10.1038/s41378-022-00369-z

**Published:** 2022-04-12

**Authors:** Fuzheng Zhang, Qijing Lin, Feng Han, Zuowei Wang, Bian Tian, Libo Zhao, Tao Dong, Zhuangde Jiang

**Affiliations:** 1 State Key Laboratory for Mechanical Manufacturing Systems Engineering, Xi’an Jiaotong University, 710049 Xi’an, China; 2 Chongqing Key Laboratory of Micro-Nano Systems and Intelligent Sensing, Chongqing Academician Workstation, Chongqing 2011 Collaborative Innovation Center of Micro/Nano Sensing and Intelligent Ecological Internet of Things, Chongqing Technology and Business University, 400067 Chongqing, China; 3 Department of Microsystems, Faculty of Technology, Natural Sciences and Maritime Sciences, University of South-Eastern Norway (USN), Raveien 215, 3184 Borre, Norway

**Keywords:** Nanosensors, Nanoscale materials

## Abstract

Flexible sensors used to detect NO_2_ gas generally have problems such as poor repeatability, high operating temperature, poor selectivity, and small detection range. In this work, a new spraying platform with a simple structure, low cost, and good film-forming consistency was designed and built to make a sensitive film (rGO/SnO_2_) for NO_2_ gas sensors. The relationship between the solid content of rGO and SnO_2_ nanoparticles, annealing temperature, and sensor performance was studied. The results show that the interdigital electrode-sensitive film formed by spraying 0.25 ml of a 0.4 wt% rGO/SnO_2_ mixture and annealing at 250 °C exhibited the best comprehensive performance for NO_2_ detection. The sensor’s response value for 100 ppm NO_2_ gas was 0.2640 at room temperature (25 °C), and the response time and recovery time were 412.4 s and 587.3 s, respectively. In the range of 20–100 ppm, the relationship between the response and NO_2_ concentration was linear, and the correlation coefficient was 0.9851. In addition, a soft-monitoring node module with an overlimit warning function for NO_2_ gas was designed and manufactured based on flexible electronics. Finally, the flexible sensor and node module were embedded into woven fabric that could be used to make a mask or a watch that could detect NO_2_ gas, realizing the practical application of flexible NO_2_ gas sensors in the field of wearable electronics.

## Introduction

Nitrogen dioxide (NO_2_) is a gas that is extremely harmful to the human body, as it can cause serious damage to lung tissues. Exposure to NO_2_ gas greatly threatens the safety of human life^1^. Therefore, real-time detection of NO_2_ gas concentration and the development of wearable NO_2_ gas detection and early warning devices for workers is of great significance, especially in environments where NO_2_ gas leakage is prone to occur, such as chemical plants.

Metal–oxide semiconductor gas sensors are widely used in gas sensing due to their high sensitivity and rapid response, and they include metal oxides such as WO_3_^2,3^, SnO_2_^4–7^, InO_3_^8,9^, and ZnO^10–12^. Using a ceramic tube as the substrate, Zhang et al. designed a gas sensor that contained SnO_2_ hollow spheres. The responses to NO_2_, methanol, acetone, NH_3_, and other gases were tested, and the results showed that NO_2_ gas had a higher response at 160 °C^13^. Wang et al. introduced a structure of P_d_-coated SnO_2_ nanofiber rods prepared by electrospinning and magnetic sputtering and used it to detect H_2_. When the hydrogen-gas concentration was 100 ppm, the detection limit of the sensor was as low as 0.25 ppm. Nevertheless, the minimum operating temperature of the sensor was also as high as 160 °C^14^. Firas et al. proposed ZnO/SnO_2_ nanorod core–shell arrays based on a quartz substrate for ethanol vapor, and the response was 0.866 at 225 °C^15^. Myadam et al. developed a formaldehyde gas sensor based on a Cu/SnO_2_ xerogel with a high response value (S = 50–96%) when the operating temperature was 325–275 °C. The sample holder of the sensing unit was made up of a ceramic sheet and two probes^16^. Most current gas sensors have rigid structures, which limits their applications^17,18^. Compared with gas sensors with rigid structures^19–21^, flexible sensors are more conducive to applications in wearable electronics because they are bendable. In addition, flexible gas sensors are easier to integrate with other flexible sensors and can be further expanded to fabricate electronic skin with multiparameter detection functions.

Most flexible NO_2_ gas sensors mainly detect low-concentration NO_2_ gas, and there have been few studies on wide-range NO_2_ gas sensors. Choi et al. have developed an integrated structure composed of a nanostructured composite sensor layer and a flexible heating substrate for detecting NO_2_. When the concentration of NO_2_ gas was in the range of 1–20 ppm, the sensor exhibited good sensing performance at 100 °C^22^. To increase the compatibility with most flexible substrates, Bernardini et al. designed an aluminum-doped zinc-oxide sensor for detecting low-concentration NO_2_ gas (0.2–2 ppm), and the operating temperature of the sensor was reduced to 100 °C^23^. Although the working temperature of most flexible NO_2_ gas sensors is lower than that of the rigid-structure gas sensors introduced above, it is still relatively high (in general, approximately 100 °C), which leads to the complexity of the sensor system and high-power consumption and is not conducive to use in wearable electronics. Therefore, it is of great significance to develop a flexible sensor that can realize wide-range NO_2_ gas detection at room temperature.

Stannic oxide (SnO_2_) is a white N-type semiconducting nanomaterial with good chemical stability. It is sensitive to a variety of reducing or oxidizing gases and is widely used to sense various gases, including those that cause environmental-pollution gases and are harmful^24^. However, SnO_2_ nanoparticles are prone to agglomeration, and this agglomeration affects the diffusion of gas molecules on the sensitive area of the sensor, and the high-temperature working conditions also limit its application^25^. Reduced graphene oxide (rGO) has both semiconductor properties and metal properties due to its unique atomic structure and complex energy-band structure, and it has good electron transfer properties^26^. In particular, its surface contains many oxygen-containing functional groups, making it quite useful in gas detection^27^. Therefore, metal–oxide nanomaterials such as SnO_2_ modified by rGO may be better able to sense gases, especially at lower operating temperatures.

In this work, because current NO_2_ gas sensors exhibit high working temperature, small detection range, and inflexibility, a flexible sensor based on polyimide (PI), interdigital electrodes, and rGO/SnO_2_ film structures was designed and fabricated for a large range of NO_2_ gas sensing at room temperature. Based on the excellent gas sensitivity of SnO_2_ and rGO, research on the adsorption of NO_2_ gas onto the sensor was carried out at different rGO/SnO_2_ nanoparticle contents and annealing temperatures. The sensing mechanism of the rGO/SnO_2_ gas sensor at room temperature has also been proposed. Moreover, a flexible gas early-warning module was proposed and designed based on flexible electronic technology and combined with a flexible gas sensor to further fabricate a wearable NO_2_ gas-detection device. The NO_2_ sensor has obvious advantages in flexibility, working temperature, and detection range.

## Experimental sections

### Materials and synthesis of rGO/SnO_2_

Graphene oxide (GO) was produced by Hummer’s method and further processed into rGO by thermal reduction^28^. SnO_2_ nanoparticles (50 nm, 99.9%) were purchased from Nanjing Jicang Nano Tech Co., Ltd. Ultrapure water was produced through an ultrapure water-manufacturing system purchased from Shanghai Hetai Instrument Co., Ltd. After 12.5 mg of rGO powder and 25 ml of ultrapure water were mixed, a uniformly dispersed 0.05 wt% rGO solution was obtained through magnetic stirring and ultrasonic dispersion. The 25 ml solution was divided equally into 5 parts, and then 10 mg, 20 mg, 30 mg and 40 mg of SnO_2_ nanoparticles were added to four of them. Finally, after ultrasonic treatment, four separate suspensions of uniformly dispersed rGO/SnO_2_ with solid rGO contents of 20 wt%, 11.1 wt%, 7.7 wt%, and 5.9 wt% were successfully obtained.

### Spraying-process platform

**Fig. 1:**
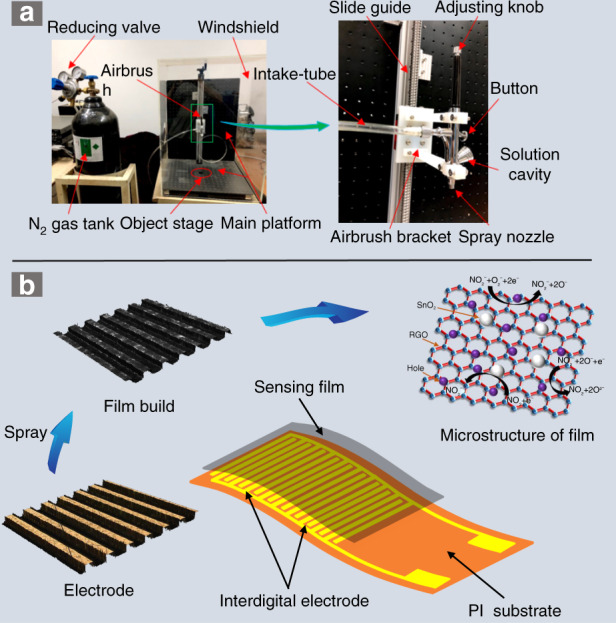
Sensor structure and fabrication platform. **a** Platform of spraying process. **b** Schematic diagram of sensor structure

The spraying-process platform shown in Fig. 1(a) has a low cost, simple structure, and convenient operation, and it includes main three parts: an air-pressure control device, an airbrush, and an operating platform. The nitrogen (N_2_) flow is controlled by regulating a pressure-reducing valve. During the spraying process, the N_2_ causes the water in the solution to evaporate and prevents it from being deposited on the interdigital electrode surface. The model of the airbrush is U-STAR-S120, and its needle diameter is 0.2 mm. The best spraying effect can be achieved by adjusting the air-flow pressure, knob of the airbrush, and height between the nozzle and the airbrush stage.

### Characterization

Scanning electron microscopy (SEM, JEOL 7800 F) was used to observe the gas sensor’s surface morphology, and an atomic force microscope (AFM, Innova) was employed to characterize the surface roughness of the sensitive film. A semiconductor-device analyzer was used to perform the I–V test of the sensor. Raman spectroscopy (HR Evolution) was performed to analyze the composition, structure and relative content of molecules to identify and characterize sensitive materials. X-ray diffraction (XRD, Bruker D8 Advance) was used to analyze the element composition and crystal structure. X-ray photoelectron spectroscopy (XPS, ESCALAB Xi+) was performed, the intensity of the photoelectron spectrum was used to characterize the content or concentration of atoms in the sensitive materials.

### Fabrication and test of the gas sensor

The flexible sensor included three main parts: the PI substrate, interdigital electrode, and sensitive rGO/SnO_2_ film. As shown in Fig. 1(b), the Cu/Au interdigital electrode was prepared on the PI substrate, the line width and line spacing were both 50 μm, and the electrode area was 5 mm × 5 mm. The overall size of the sensor is 7 mm × 11 mm. The mixed solution was deposited on the surface of the interdigital electrode through a spraying platform and then dried in a vacuum-drying oven (60 °C, 1 h) to form a gas-sensitive film. A high-temperature annealing furnace was used to anneal the sensitive film, and N_2_ was used as the protective gas. A digital multimeter (Keysight, 34461 A) was used to record the resistance of the sensor. A gas-flow quality controller (Sevenstar, CS200) was applied to adjust the NO_2_ gas concentration, and the mixed gas was dry air. The response (S) is defined as |*R*_0_–*R*_a_|/*R*_0_, where *R*_0_ is the sensor’s resistance in air and *R*_a_ is the resistance in NO_2_ gas. The response time (*t*_res_) is defined as the time when the sensor reaches 90% of the total response in a NO_2_ gas environment, and the recovery time (*t*_rec_) is the time when the sensor returns to 10% of the total response in the air. The temperature of the gas-test environment was 25 °C.

## Results and discussion

### Sensing performance of the rGO film and characterization of the film

**Fig. 2:**
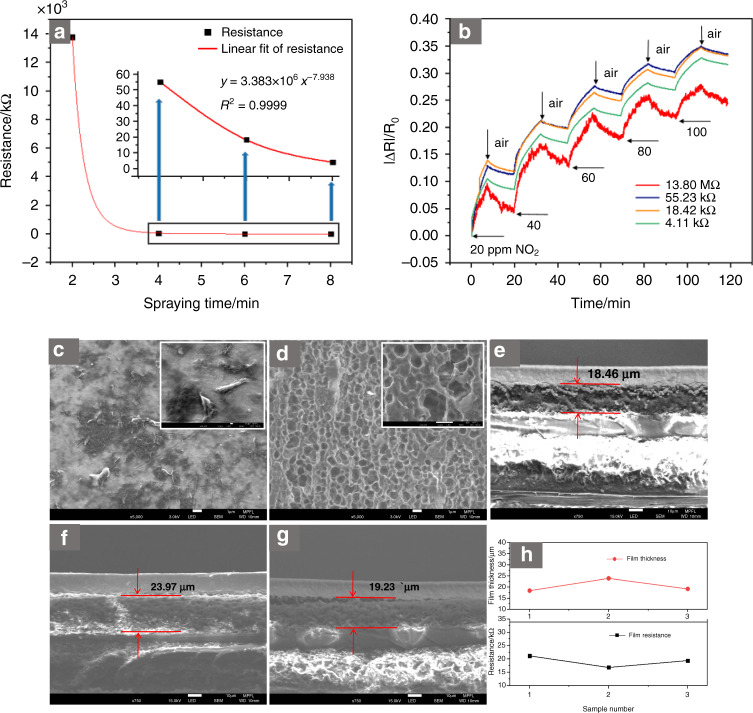
Gas-sensing performance and film-forming consistency test results of rGO thin films. **a** Resistance of sensors with different rGO contents. **b** Response curves of sensors with different rGO contents toward different NO_2_ concentrations, SEM images of rGO film, (**c**) electrode surface, (**d**) PI surface (groove), (**e**), (**f**), and (**g**) rGO film thickness of three sprays, and (**h**) resistance and thickness curves of three sprays

In the spraying process, the rGO content in the film was controlled by the spraying time. Four sensors with different rGO contents were constructed in this study, and the spraying time was 2, 4, 6, or 8 min, depending on the sample. The sensors’ resistance decreased with increasing rGO content, showing a trend of exponential decay, which decreased sharply before 4 min and slowed after 4 min, as shown in Fig. 2(a). The response curves of sensors with different rGO contents for NO_2_ gas are shown in Fig. 2(b). The results reveal that the response increased first and then decreased with increasing rGO content, with the highest response between 4 and 6 min of spraying. The response curve had much noise, as the material was not smooth after 2 min of spraying. This roughness occurred because the interdigital electrode was not completely covered by the low-content rGO film, so the film performance was unstable.

I–V characteristic curves of the sensors with different rGO contents were also tested and are shown in Fig. 9 (Supplementary information). The conductivity of the sensor increased with increasing rGO content, and the linear-fitting coefficients were 0.9931, 0.9986, 0.9993, and 0.9996. After many experiments, it has been confirmed that the rGO film formed by spraying 0.25 ml of 0.05 wt% rGO solution has better NO_2_ gas sensitivity. However, regardless of the rGO content, the recovery time of the pure rGO sensor was very long, and it was difficult for the sensor to recover to its initial state, which is determined by the characteristics of rGO itself.

SEM images of the rGO film are shown in Fig. 2(c, d). When the morphology of the rGO film on the surface of the interdigital electrode is compared with that on the PI-groove surface, it is observed that there are many circular holes on the PI groove, the film on the groove surface is far less flat than that on the interdigital electrode surface. The reason for this phenomenon is that the surface roughness of the interdigital electrode (R_a_ = 26.6 nm) is less than that of the PI-groove surface (R_a_ = 142 nm) before the rGO solution was sprayed, as shown in Fig. 10(b) and Fig. 10(c) (Supplementary information), respectively. The roughness of the PI-substrate surface outside the interdigital electrode area is 5.44 nm, as shown in Fig. 10(a) (Supplementary information), which indicates that the stripping process increases the roughness of the PI-groove surface during preparation.

Figure 2(e), (f) and (g) are cross-sectional images of rGO films. The films were all made through the same process, and each spraying solution had a volume of 0.25 ml. The thicknesses of the three films were 18.46 μm, 23.97 μm, and 19.23 μm, and the corresponding resistance values were 21.23 kΩ, 16.87 kΩ, and 19.45 kΩ, respectively. As shown in Fig. 2(h), the film’s thickness was inversely proportional to its resistance value. The resistance of the pure rGO film prepared by the spraying method was stable between 10 and 30 kΩ. In addition, Fig. 10(d) and (g), (e) and (h), (f) and (i) (Supplementary information) shows the roughness of the rGO film on the interdigitated electrode surface and the PI-groove surface of the three sensors, respectively. One was mostly stable at approximately 30 nm, and the other was mostly stable at approximately 180 nm; these results show that the spraying-process platform has good film-forming consistency.

### Sensing performance of the rGO/SnO_2_ sensor

**Fig. 3:**
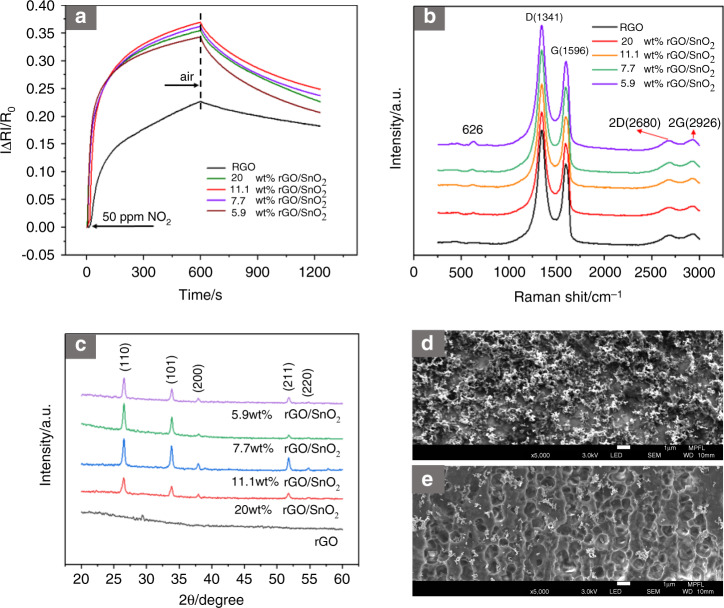
Response curve and characterization results for different concentrations of rGO/SnO_2_. **a** The response curve at 50 ppm NO_2_, (**b**) Raman spectra, (**c**) XRD pattern, and SEM images of the (**d**) electrode surface and (**e**) PI surface (groove) of the sensor with 11.1 wt% rGO/SnO_2_

The response curve of the sensors doped with different contents of SnO_2_ nanoparticles is shown in Fig. 3(a), and the response was the highest at 11.1 wt% rGO/SnO_2_. Figure 3(d, e) shows SEM images of the 11.1 wt% rGO/SnO_2_ film. The proper amount of SnO_2_ nanoparticles increased the surface roughness of the film and provided more active sites for NO_2_ gas, which was beneficial for improving the response of the sensor. Although the recovery time of the rGO/SnO_2_ film was improved to a certain extent, it was still difficult to fully recover. In Fig. 3(b), the Raman spectra of the rGO film and the rGO/SnO_2_ film both have obvious D (1341 cm^−1^) and G (1596 cm^−1^) peaks of rGO. The D peak represents the rGO edge defects, and the G peak is generated by the stretching motion of the sp^2^ atom pairs. The 2D peak appears near 2680 cm^−1^ and is caused by two-phonon resonance, which is closely related to the number of rGO layers. The 2G peak appears near 2926 cm^−1^, and it is also characteristic of rGO. The weak peak at 626 cm^−1^ is caused by the vibration of SnO_2_, which indicates that the rGO/SnO_2_ film contains SnO_2_ particles. In Fig. 3(c), the characteristic peaks are located at 2θ = 26.528°, 33.811°, 37.910°, 51.723°, and 54.800°, which are consistent with the (110), (101), (200), (211), and (220) planes of the tetragonal rutile structure of SnO_2_ (Jcpds. No. 41-1445).

**Fig. 4:**
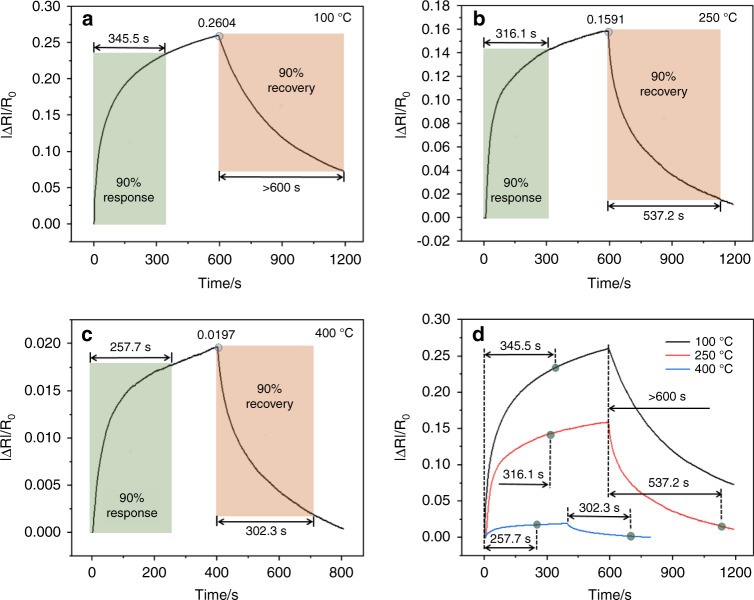
The response-change curves of the sensor with 11.1 wt% rGO/SnO_2_ for 50 ppm NO_2_ rGO/SnO_2_ at different annealing temperatures. **a** 100 °C, (**b**) 250 °C, (**c**) 400 °C, and (**d**) curves of three annealing temperatures

To further improve the sensor recovery performance, annealing was carried out, as shown in Fig. 4. The annealing temperatures were 100 °C, 250 °C, and 400 °C. N_2_ was used as the protective gas, and the annealing time was 1 h. When the annealing temperature was 100 °C, the response of the sensor was lower than that of the control sample. Although the recovery performance was improved to a certain extent, the recovery time was still long (over 600 s). The response of the sensor, response time, and recovery time were 0.1591, 316.1 s, and 537.2 s, respectively, at 50 ppm NO_2_. Although the response decreased, the response time and recovery time improved, and recovery fully occurred. As the annealing temperature increased to 400 °C, although the response-time and recovery-time performance significantly improved, the response of the sensor also decreased sharply to 0.0197. In summary, annealing affected the performance of the sensor for NO_2_ gas, and this effect specifically manifested in the lower response and shortened response time and recovery time. When the annealing temperature was 250 °C, the sensor showed the best performance.

**Fig. 5:**
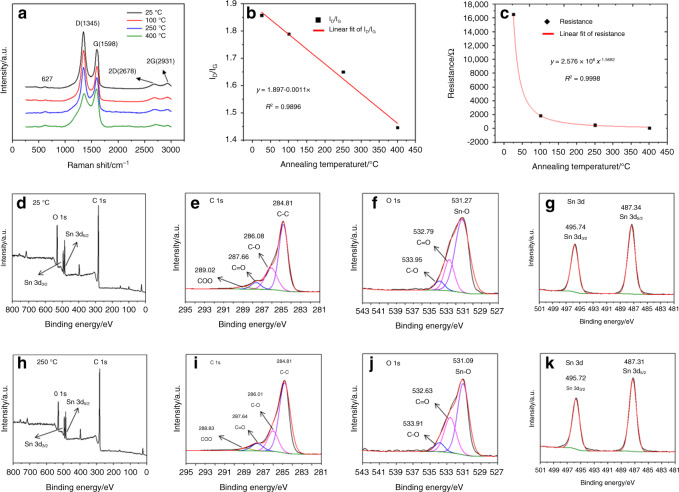
In all, 11.1 wt% rGO/SnO_2_ sensor’s characterization results. **a** Raman spectra, (**b**) change in I_D_/I_G_, (**c**) change in the resistance at different annealing temperatures, XPS spectra of (**d**) rGO/SnO_2_, (**e**) C 1 s spectrum, (**f**) O 1 s spectrum and (**g**) Sn 3d spectrum at room temperature (25 °C), XPS spectra of (**h**) rGO/SnO_2_, (**i**) C 1 s spectrum, (**j**) O 1 s spectrum and (**k**) Sn 3d spectrum of the sample annealed at 250 °C

Raman spectra of the sensor with 11.1 wt% rGO/SnO_2_ at different annealing temperatures are shown in Fig. 5(a). The results show obvious characteristic peaks of rGO and SnO_2_ after annealing, which implies that annealing does not change the sensitive material of the sensor. The I_D_/I_G_ ratio reflects the number of defects in rGO, the larger the ratio is, the more defects are present. As the annealing temperature was increased, I_D_/I_G_ decreased, as shown in Fig. 5(b), which indicates that the number of rGO defects decreased. Therefore, there were fewer active sites on the sensitive-film surface, which reduced the sensor’s response to NO_2_ gas. In addition, the reduction in rGO defects decreased the resistance of the sensitive film, as shown in Fig. 5(c).

The wide-range XPS spectrum of the rGO/SnO_2_ sensor (without annealing) is shown in Fig. 5(d). The main peaks correspond to C, O, and SnO_2_, and these peaks indicate that the film was not doped with other impurities during preparation. As shown in Fig. 5(e), four peaks of the C 1 s spectrum centered at 289.02, 287.66, 286.08, and 284.81 eV are observed, corresponding to COO, C=O, C–O, C–C and groups, respectively. C–C has the highest proportion (62.65%), which shows that rGO was successfully prepared by thermal reduction. Figure 5(f) shows the high-resolution spectrum of the O 1 s orbitals. Three characteristic peaks appear at binding energies of 531.27 eV, 532.79 eV, and 533.95 eV, corresponding to Sn–O, C=O, and C–O, respectively. Figure 5(g) shows the spectrum of Sn 3d. Two characteristic peaks appear at 487.34 eV and 495.74 eV, corresponding to the Sn 3d_3/2_ and Sn 3d_5/2_ orbitals, respectively. The oxidation state of Sn remained 4+, so the SnO_2_ did not change during the material-modification process. In addition, in contrast to the peaks of the unannealed rGO/SnO_2_ film, the characteristic peaks of the elements in the rGO/SnO_2_ film after annealing barely changed, which shows that annealing does not affect the properties of the mixed material. However, the proportion of C–C bonds was 69.97% in Fig. 5(i), which was higher than that of the C–C bonds in the unannealed samples. This result indicates that rGO was further reduced during the annealing of the rGO/SnO_2_ film, which led to the decrease in resistance of the rGO/SnO_2_ film.

A P–N junction can be formed between N-type SnO_2_ and P-type rGO. To study the effects of the annealing process on the P–N junction, the Hall effect of the two sensors after annealing was tested, as shown in Table 1 (Supplementary information). The sensitive films of the two sensors were pure rGO and rGO/SnO_2_. At the same annealing temperature (250 °C), the test results show that the Hall mobility of the rGO/SnO_2_ film was greater than that of the pure rGO film, and its corresponding sheet carrier concentration was less than that of the rGO film, which shows that annealing activates the P–N junction. The P–N junction can effectively increase the electron transmission rate, which is the main reason for the improved recovery time of the sensor.

**Fig. 6:**
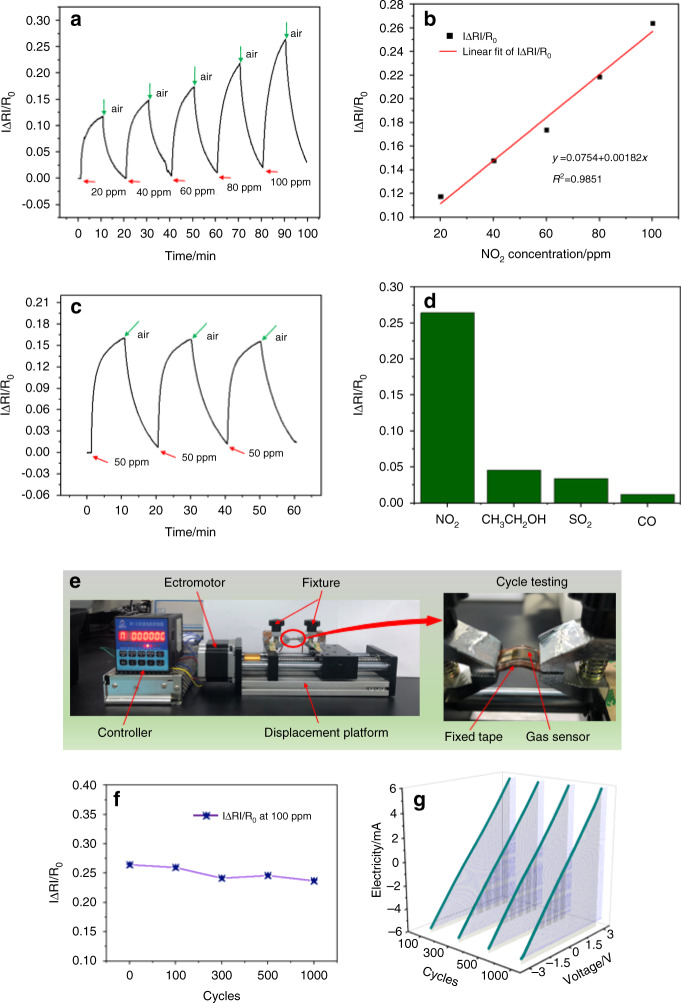
Gas-sensing performance test results of the sensor. **a** Response of the 11.1 wt% rGO/SnO_2_ sensor (sample annealed at 250 °C) in the range of 20–100 ppm NO_2_, (**b**) linear fit curve of |*R*_0_–*R*_a_|/*R*_0_, (**c**) repeatability at 50 ppm NO_2_, (**d**) gas selectivity at 100 ppm, (**e**) flexibility test platform for gas sensor, (**f**) response curve and (**g**) I–V curve of different cycles

Figure 6(a) shows the relationship between the response of the rGO/SnO_2_ sensor and the NO_2_ gas concentration. In the concentration range of 20–100 ppm, the response increased with increasing NO_2_ concentration; these values were 0.1176 (20 ppm), 0.1480 (40 ppm), 0.1738 (60 ppm), 0.2186 (80 ppm), and 0.2640 (100 ppm), respectively. The response and recovery times of the sensor when sensing 100 ppm NO_2_ were 412.4 s and 587.3 s, respectively. The relationship between the response and the NO_2_ concentration was linear, and the linear correlation was 0.9851, as shown in Fig. 6(b). Figure 6(c) shows the repeatability of the rGO/SnO_2_ sensor at 50 ppm NO_2_. The sensor’s performance parameters, such as response, response time, and recovery time, did not drift over three cycles, showing good stability, therefore, this sensor can be used for continuous gas monitoring. The sensor’s selectivity for different gases (NO_2_, CH_3_CH_2_OH, SO_2_, and CO) is shown in Fig. 6(d), and it is confirmed that the sensor has good selectivity for NO_2_ gas.

The flexible sensor testing platform is shown in Fig. 6(e). The responses toward 100 ppm NO_2_ after 100, 300, 500, and 1000 cycles were 0.2596, 0.2413, 0.2459 and 0.2367, respectively. As shown in Fig. 6(f), compared with the response value before cyclic bending, that after bending was slightly lower. It remained above 90% of the initial value, which shows that the sensor had good mechanical properties. Figure 6(g) shows the I–V characteristics of the sensor after cycling, and the result shows that the change in the sensor current was almost negligible. All these results indicate that the sensor is flexible and is applicable to wearable electronics.

**Fig. 7:**
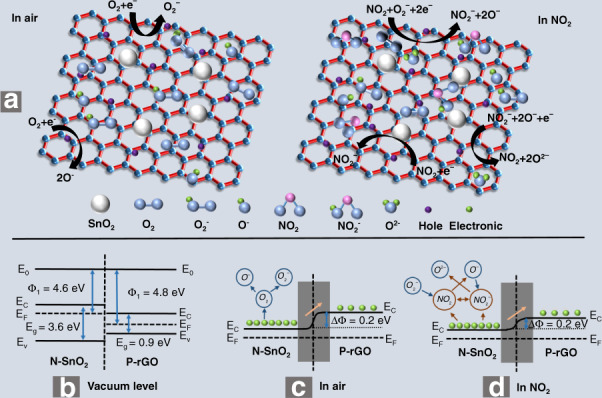
Schematic diagram of gas sensing mechanism for rGO/SnO_2_-based sensor toward NO_2_ gas. **a** The gas-sensing mechanism, the working functions of rGO and SnO_2_ in (**b**) vacuum level, (**c**) air, (**d**) NO_2_

For NO_2_ gas sensing, the rGO/SnO_2_ composite material showed the characteristics of a P-type semiconductor, so the rGO dominated the conduction channel. The excellent sensing performance of the rGO/SnO_2_ sensor mainly depends on the P–N junction. The gas-sensing mechanism is shown in Fig. 7(a). Oxygen adsorption plays a very important role in the charge-transfer process of the rGO/SnO_2_ composite^29^. When the sensor is exposed in air, oxygen molecules are adsorbed on the SnO_2_ surface and become negative oxygen ions, forming a depletion layer. The process of forming oxygen-negative ions is shown in Eqs. (1)–(3):

1 $${\rm{O}}_2({\rm{gas}}) \to {\rm{O}}_2({\rm{ads}})$$

2 $${\rm{O}}_{^2}({\rm{ads}}) + e^ - \to {\rm{O}}_{^2}^ - ({\rm{ads}})$$

3 $${\rm{O}}_{^2}^ - ({\rm{ads}}) + e^ - \to 2{\rm{O}}^ - ({\rm{ads}})$$

As shown in Fig. 7(b), under vacuum, the working functions of rGO and SnO_2_ are 4.8 eV and 4.6 eV, respectively^30^. Therefore, to balance the Fermi level, electrons are transferred from SnO_2_ to rGO, which forms a depletion layer and a heterojunction barrier, as shown in Fig. 7(c). When the sensor is exposed to an NO_2_ gas environment, NO_2_ has a higher electronegativity than the oxygen molecules. More electrons are deprived from the surface of oxygen and rGO/SnO_2_. The reaction processes are shown below:

4 $${\rm{NO}}_{^2}({\rm{gas}}) \to {\rm{NO}}_{^2}({\rm{ads}})$$

5 $${\rm{NO}}_{^2}({\rm{ads}}) + e^ - \to {\rm{NO}}_2^ - ({\rm{ads}})$$

6 $${\rm{NO}}_{^2}({\rm{ads}}) + {\rm{O}}_2^ - ({\rm{ads}}) + 2e^ - \to {\rm{NO}}_2^ - ({\rm{ads}}) + 2{\rm{O}}^ - ({\rm{ads}})$$

7 $${\rm{NO}}_2^ - ({\rm{ads}}) + 2{\rm{O}}^ - ({\rm{ads}}) + e^ - \to {\rm{NO}}_{^2}({\rm{gas}}) + 2{\rm{O}}^{2 - }({\rm{ads}})$$

During this process, as shown in Fig. 7(d), the conduction band in the rGO/SnO_2_ composite material consumes more electrons. The corresponding hole concentration increases, which reduces the resistance of the sensor. In addition, the Fermi level of SnO_2_ is far from the conduction band, which leads to the lowering of the heterojunction barrier. In addition, the introduction of SnO_2_ nanoparticles provides more active sites, such as defects and oxygen-containing functional groups, that can adsorb more NO_2_ gas on the surface of rGO. In short, the P–N heterostructure formed between SnO_2_ and rGO effectively accelerates the electron-transfer rate and improves the sensing performance of NO_2_ gas by changing its own resistance.

### Early-warning module and wearable applications

The circuit principle of the monitoring node module is shown in Fig. 11 (Supplementary information), which includes a booster circuit, a main control-chip circuit, a debug serial-port circuit, two ADC-detection circuits, and three LED-light circuits. The functions of each part are as follows:

1. Booster circuit: This circuit converts the 3 V voltage output by the button battery to 5 V to meet the power-supply requirement of the node module.
2. Main control-chip circuit: The STC chip is used as the main control chip to control and manage the entire early-warning system.
3. Debug serial-port circuit: The program instructions are input into the main control chip.
4. ADC-detection circuit: The resistance of the sensor is detected to determine whether it exceeds the threshold.
5. LED-light circuit: This circuit is used to judge whether the NO_2_ gas concentration exceeds the preset detection threshold through its blinking.

The size of the monitoring-node module was d = 3 cm, and the thickness of the base was approximately 0.2 mm, as shown in Fig. 12(a, b) (Supplementary information). The front side and back side of the monitoring node module are shown in Fig. 12(c, d) (Supplementary information), respectively. A button battery was placed on the back side of the node module. There were two sensor interfaces: one was used to connect the NO_2_ sensor, and the other was used as a spare interface. One of the warning LED lights was red, and the other was green. In this way, even if one interface were to become damaged, it would not affect the normal operation of the node module.

**Fig. 8:**
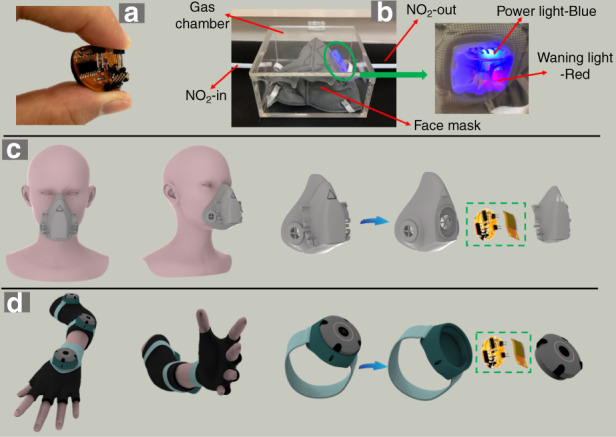
Overlimit warning testing of sensor system and wearable applications. **a** Bending test of node module, (**b**) over-limit warning for NO_2_ gas, and wearable applications for (**c**) masks and (**d**) watches

The monitoring-node module has good flexibility and can be bent at a large angle, as shown in Fig. 8(a). The system’s overlimit warning-test results are shown in Fig. 8(b). When the NO_2_ gas concentration exceeds the preset threshold (20 ppm in this test), the red warning indicator light turns on. The blue LED light is used as the power light to indicate that the system power is normal. Therefore, the node module and rGO/SnO_2_ sensor have wide applications in the wearable field because they are small and flexible. They can be embedded in masks and watches, as shown in Fig. 8(c, d).

Compared with the other NO_2_ gas sensors reported in Table 2 (Supplementary information), this sensor exhibited better performance at room temperature and has significant advantages in performance indicators such as response and flexibility. The flexible sensor has a wider linear NO_2_-detection range and a shorter response time and recovery time for highly concentrated NO_2_ gas, which greatly broaden the application of this sensor. In addition, combined with the designed flexible early-warning module, a wearable electronic device with a wide detection range and an early-warning function for NO_2_ was demonstrated in this paper.

## Conclusion

In summary, an rGO/SnO_2_ sensor developed via spraying to form an interdigital electrode structure was proposed and fabricated to detect a wide range of NO_2_ gas concentrations. It had the advantages of a high response and selectivity and a short response time and recovery time. The sensor substrate was PI, which has good flexibility. In addition, a spraying platform with a simple structure, low cost, and good film-forming consistency was designed and built, which enabled the manufacture of small-batch sensors. The relationship between the sensor’s response and the NO_2_ concentration was linear in the range of 20–100 ppm NO_2_, and the linear correlation was 0.9851, which shows that the sensor had good performance. Moreover, a flexible monitoring-node module based on soft-board technology was designed and completed, achieving the overlimit warning function of NO_2_ gas. Finally, a wearable application of the flexible sensor system was demonstrated by embedding the flexible-node module and the sensor into a mask and a watch, and it therefore showed great application potential in wearable electronics.

## Supplementary information

Supplementary Information
