# Supplementary material for: A flexible and wearable NO2 gas detection and early warning device based on a spraying process and an interdigital electrode at room temperature
Source: Microsyst Nanoeng. 2022 Apr 12;8:40. doi: 10.1038/s41378-022-00369-z (PMC9005537; doi:10.1038/s41378-022-00369-z)
Supplement: Supplementary file 1 — Supplementary Information [file 41378_2022_369_MOESM1_ESM.docx]

| 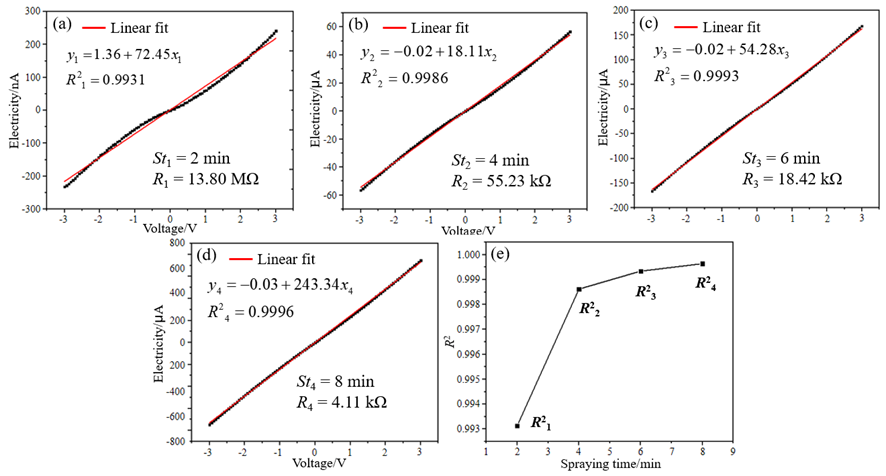 |
| --- |
| **Fig. 9.** I-V characteristic curves of sensors with different rGO content. (a) *S*_t1_ = 2 min, (b) *S*_t2_ = 4 min, (c) *S*_t3_ = 6 min, (d) *S*_t4_ = 8 min, (e) Fitting coefficient curve. |
| 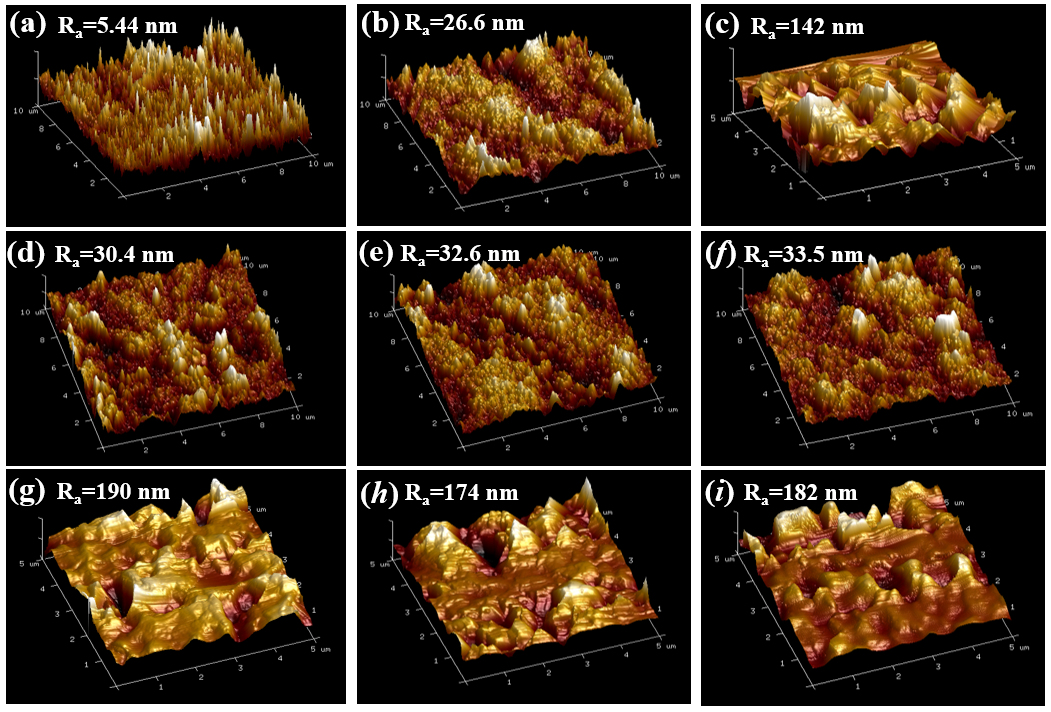 |
| **Fig. 10.** AFM images. (a) PI, (b) Interdigital electrode, (c) PI (Groove). (d) and (g), (e) and (*h*), (*f*) and (*i*), the electrode surface and PI (Groove) surface of the rGO films formed after three sprays. |

**Table 1** Hall effect test results of the sensor

| Sensitive film | Hall mobility (cm^2^/V‧s) | Sheet carrier concentration (1/cm^2^) | Annealing temperature(℃) |  |
| --- | --- | --- | --- | --- |
| rGO | 31.426 | 1.0192×10^19^ | 250 |  |
| rGO/SnO_2_ | 46.912 | 6.4747×10^18^ | 250 |  |
| 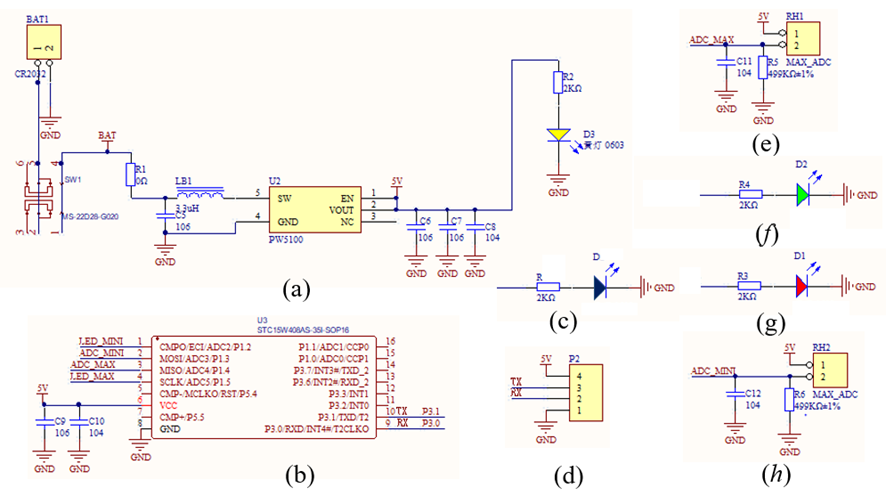 | | | | |
| **Fig. 11.** Circuit schematic diagram of monitoring node module. (a) booster circuit, (b) main control chip, (c) power light circuit , (d) Debug serial port circuit, (e) ADC detection circuit 2, (*f*) LED light circuit 2, (g) LED light circuit 1, (*h*) ADC detection circuit 1. | | | | |
| 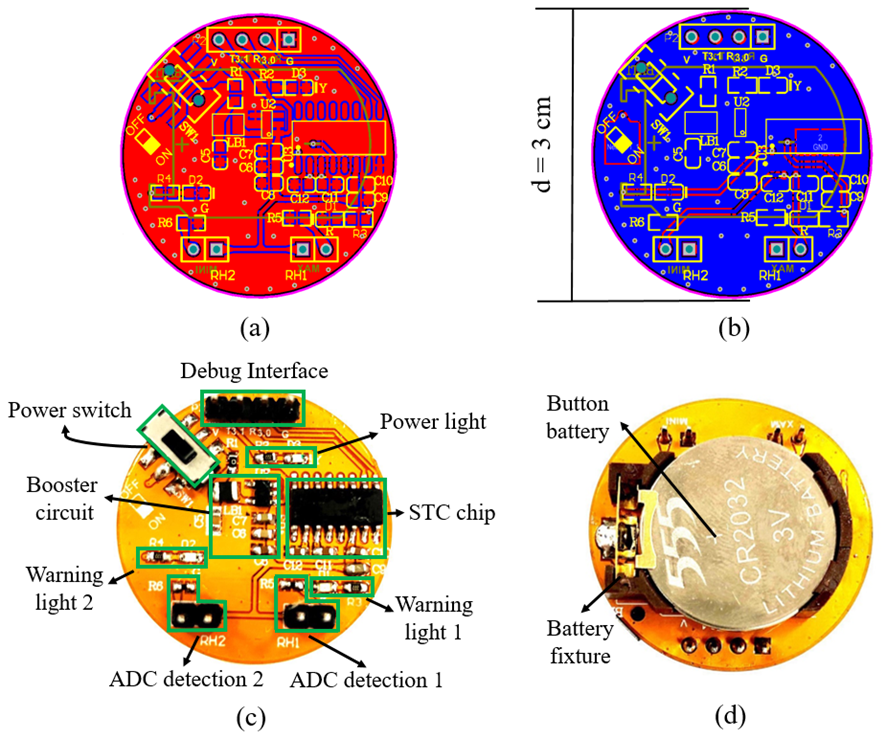 | | | | |
| **Fig. 12.** Monitoring node module. PCB of (a) front side and (b) back side, profile display of (c) front side and (d) back side. | | | | |

**Table 2** Comparison of various gas-sensing materials towards NO_2_ gas in previous literature

| Material | Method | NO_2_  (ppm) | Temp.  (℃) | S | T_res_/t_rec_  (s) | Range  (ppm) | linearity | Early  warning | flexibility | Ref. |
| --- | --- | --- | --- | --- | --- | --- | --- | --- | --- | --- |
| SnO_2_/rGO | Hydrothermal process and drip coating process | 0.75 | 116 | 0.136 | 16.8/90 | 0.05-1 | 0.987 |  |  | [31] |
| (3D) SnO_2_/rGO | sol–gel method | 100 | 55 | 0.08 | -/373 | 14-110 | 0.993 |  |  | [32] |
| rGO/ZnO | Wet spinning and hydrothermal treatment | 1.5 | RT | 0.014 | 405/760 | 1.5-19.4 |  |  |  | [33] |
| Ag-rGO | Hydrothermal process and spraying process | 5 | RT | 0.056 | 990/>1566 | 5-50 | 0.9495 |  | Good | [34] |
| ZnO/GA_S_ | Solvothermal route | 50 | RT | 0.08 | 132/164 | 10-200 |  |  |  | [35] |
| rGO/SnO_2_ | Hydrothermal process and spraying process | 25 | RT | 0.22 |  | 10-25 | 0.99 |  |  | [36] |
| rGO/SnO_2_ | Hydrothermal process, Spraying process and annealing treatment | 100 | RT | 0.264 | 412.4/587.3 | 20-100 | 0.9851 | Yes | Good | This work |

[31] Wang Y, Liu L, Sun F, et al. Humidity-Insensitive NO_2_ Sensors Based on SnO_2_/rGO Composites[J]. Frontiers in Chemistry, 2021, 9:681313. 10.3389/fchem.2021.681313

[32] Li L, He S, Liu M, et al. Three-dimensional mesoporous graphene aerogel-supported SnO_2_ nanocrystals for high-performance NO_2_ gas sensing at low temperature.[J]. Analytical Chemistry, 2015, 87(3):1638-1645. 10.1021/ac503234e

[33] Adu A, Ggu B, Shj C, et al. ZnO decorated flexible and strong graphene fibers for sensing NO_2_ and H_2_S at room temperature[J]. Sensors and Actuators B: Chemical, 308. 10.1016/j.snb.2020.127690

[34] Li C, Li W W, Cai L, et al. Flexible nitrogen dioxide gas sensor based on reduced graphene oxide sensing material using silver nanowire electrode[J]. Acta Physica Sinica -Chinese Edition, 2020, 69(5):058101. 10.7498/aps.69.20191390

[35] Liu X, Sun J, Zhang X. Novel 3D graphene aerogel–ZnO composites as efficient detection for NO_2_ at room temperature[J]. Sensors & Actuators B Chemical, 2015, 211:220-226. 10.1016/j.snb.2015.01.083

[36] Zhu X, Guo Y, Hao R, et al. Enhancing the NO_2_ gas sensing properties of rGO/SnO_2_ nanocomposite films by using microporous substrates[J]. Sensors & Actuators B Chemical, 2017, 248:560-570. 10.1016/j.snb.2017.04.030
